# Practical Notes on Diagnosis and Treatment

**Published:** 1907-12-07

**Authors:** 


### Practical Notes on Diagnosis and Treatment.

**Gonorrhœal Rheumatism.**

Salol and sandal wood oil are reported to give good results in this disease. They may be ordered in capsules.

**Earache in Children.**

In all cases of indefinite pain in the head in young children the tympanic membrane should be examined; these patients are often quite unable to localise the pain of earache.

**Typhoid Bacilli in the Urine.**

The bacilli in typhoid fever may be found in the urine as well as in the fæces. Ten grains of hexamethylene tetramine three times a day will be found efficacious in sterilising the urine.

**Gastric Crises.**

Attacks of gastric pain and vomiting may be evidences of tabes dorsalis, even though the patient has no other symptoms of the disease. Hence, in such circumstances a careful note should be made of the pupils and knee-jerks. Sometimes the gastric symptoms include hæmatemesis.

**Whooping Cough.**

The paroxysmal seizures of whooping cough are much relieved by syringing the ears night and morning with warm boric lotion, this on each occasion being followed by painting the external meatus and tympanic membrane with a solution of cocaine and perchloride of mercury.—*Dr. G. A. Stephens.*

**Belladonna in Broncho-pneumonia.**

Broncho-pneumonia is a very fatal disease in early life, and treatment generally is unsatisfactory. Dr. J. A. Coutts strongly urges the usefulness of belladonna, which probably acts by checking pulmonary and bronchial secretion. He prescribes the extract in quarter-grain doses repeated every three or four hours. This dose should be given whatever the age of the child. Beyond slight delirium there are no toxic results.

**The Complications of Mumps.**

As rare complications or after-effects of mumps, iritis, paralysis of accommodation, and optic nerve atrophy have been noted; multiple peripheral neuritis has also been recorded.

**Ocular Palsies in Diphtheria.**

Though paralysis of accommodation is the most frequent muscular disturbance in the eyeball produced by diphtheria, the disease does sometimes cause paresis of one or other of the external ocular muscles.

**Renal Typhoid.**

Several cases have been reported in which pyrexia and hæmaturia were the sole symptoms of an attack of typhoid. Widal's test was positive, and the urine contained large numbers of the bacillus typhosus.

**Quinine in Leucorrhœa.**

Quinine ordered in the form of a pessary—three to five grains of the hydrochloride is a suitable dose—is often successful in the treatment of leucorrhœa in the female. A pessary should be introduced every night at bedtime.

**Foreign Bodies beneath the Nails.**

To remove a foreign body which has become embedded beneath the nail apply a 10 per cent. solution of caustic potash to soften the nail, and scrape away the softened portion. Repeat this until the foreign body is exposed and can be removed.

**Functional Heart Disease.**

Patients with functional derangements of the heart, often to their own hurt, make them an excuse for avoiding exercise and for taking stimulants. If any cardiac symptom can be "walked off" it may be put down as functional, as may also an intermittent action of the pulse of which the patient is conscious. In women angina pectoris is nearly always functional.—*Sir William H. Broadbent.*

